# Autocycler: long-read consensus assembly for bacterial genomes

**DOI:** 10.1093/bioinformatics/btaf474

**Published:** 2025-08-28

**Authors:** Ryan R Wick, Benjamin P Howden, Timothy P Stinear

**Affiliations:** Department of Microbiology and Immunology, The University of Melbourne at the Peter Doherty Institute for Infection and Immunity, Melbourne, VIC, 3000, Australia; Centre for Pathogen Genomics, The University of Melbourne, Parkville, VIC, 3010, Australia; Department of Microbiology and Immunology, The University of Melbourne at the Peter Doherty Institute for Infection and Immunity, Melbourne, VIC, 3000, Australia; Centre for Pathogen Genomics, The University of Melbourne, Parkville, VIC, 3010, Australia; Department of Microbiology and Immunology, The University of Melbourne at the Peter Doherty Institute for Infection and Immunity, Melbourne, VIC, 3000, Australia; Centre for Pathogen Genomics, The University of Melbourne, Parkville, VIC, 3010, Australia

## Abstract

**Motivation:**

Long-read sequencing enables complete bacterial genome assemblies, but individual assemblers are imperfect and often produce sequence-level and structural errors. Consensus assembly using Trycycler can improve accuracy, but its lack of automation limits scalability. There is a need for an automated method to generate high-quality consensus bacterial genome assemblies from long-read data.

**Results:**

We present Autocycler, a command-line tool for generating accurate bacterial genome assemblies by combining multiple alternative long-read assemblies of the same genome. Without requiring user input, Autocycler builds a compacted De Bruijn graph from the input assemblies, clusters and filters contigs, trims overlaps, and resolves consensus sequences by selecting the most common variant at each locus. It also supports manual curation when desired, allowing users to refine assemblies in challenging or important cases. In our evaluation using Oxford Nanopore Technologies reads from five bacterial isolates, Autocycler outperformed individual assemblers, automated pipelines, and other consensus tools, producing assemblies with lower error rates and improved structural accuracy.

**Availability and implementation:**

Autocycler is implemented in Rust, open-source, and freely available at github.com/rrwick/Autocycler. It runs on Linux and macOS and is extensively documented.

## 1 Introduction

Complete genome assemblies are essential for resolving bacterial genome structure and fully characterizing accessory elements such as plasmids and prophages (Decano *et al.* 2019, Gulliver *et al.* 2023). Accurate assemblies reduce the risk of errors in downstream analyses such as comparative genomics, annotation, and studies of genome dynamics.

Long-read sequencing platforms, such as those from Oxford Nanopore Technologies (ONT), have made complete assemblies of bacterial genomes widely achievable. Long reads can span repetitive elements, allowing assemblers to resolve structural complexity that short reads (e.g. from Illumina platforms) cannot (Koren *et al.* 2013). For most bacterial genomes and high-quality read sets, long-read assemblers can assemble each replicon into a single contig (Wick and Holt 2019).

In practice, however, long-read assemblers are imperfect, and different tools produce different assemblies from the same input read set. Common problems include: incomplete or overlapping circularization, missing small plasmids, duplicated small plasmids, and spurious extra contigs from repeats or contamination (Boostrom *et al.* 2022, Johnson *et al.* 2023). No single assembler is reliably the best across all datasets.

Consensus assembly offers a solution. By combining multiple alternative assemblies of the same genome (e.g. those produced by different assemblers or read subsets), consistent sequences can be distinguished from assembler-specific errors (Lin and Liao 2013, Wences and Schatz 2015). The software Trycycler put this idea into practice for bacterial genomes (Wick *et al.* 2021). Compared to assemblies produced by a single tool, Trycycler assemblies usually contain fewer errors, more reliable circularization, and a more complete and less contaminated representation of the genome (Wick *et al.* 2021).

Trycycler, however, relies on human interventions and decision-making for several key steps. While this design offers flexibility and control, it limits scalability. As bacterial genomics increasingly involves large datasets of hundreds or thousands of genomes, there is a need for automated methods that can generate high-quality consensus assemblies without manual interventions.

Here, we present Autocycler, an automated command-line tool to generate consensus long-read assemblies of bacterial genomes. Like Trycycler, it combines multiple input assemblies to produce a high-quality consensus. Unlike Trycycler, Autocycler is designed to run to completion without user input. It also supports manual intervention for cases where careful output curation is warranted. For most bacterial genomes and read sets with sufficient depth and read length, Autocycler can produce complete assemblies automatically. In more difficult cases, such as genomes with large repeats, genomic heterogeneity or unusual structures like linear replicons, users can step in to refine the output.

## 2 Implementation

**Figure 1.**
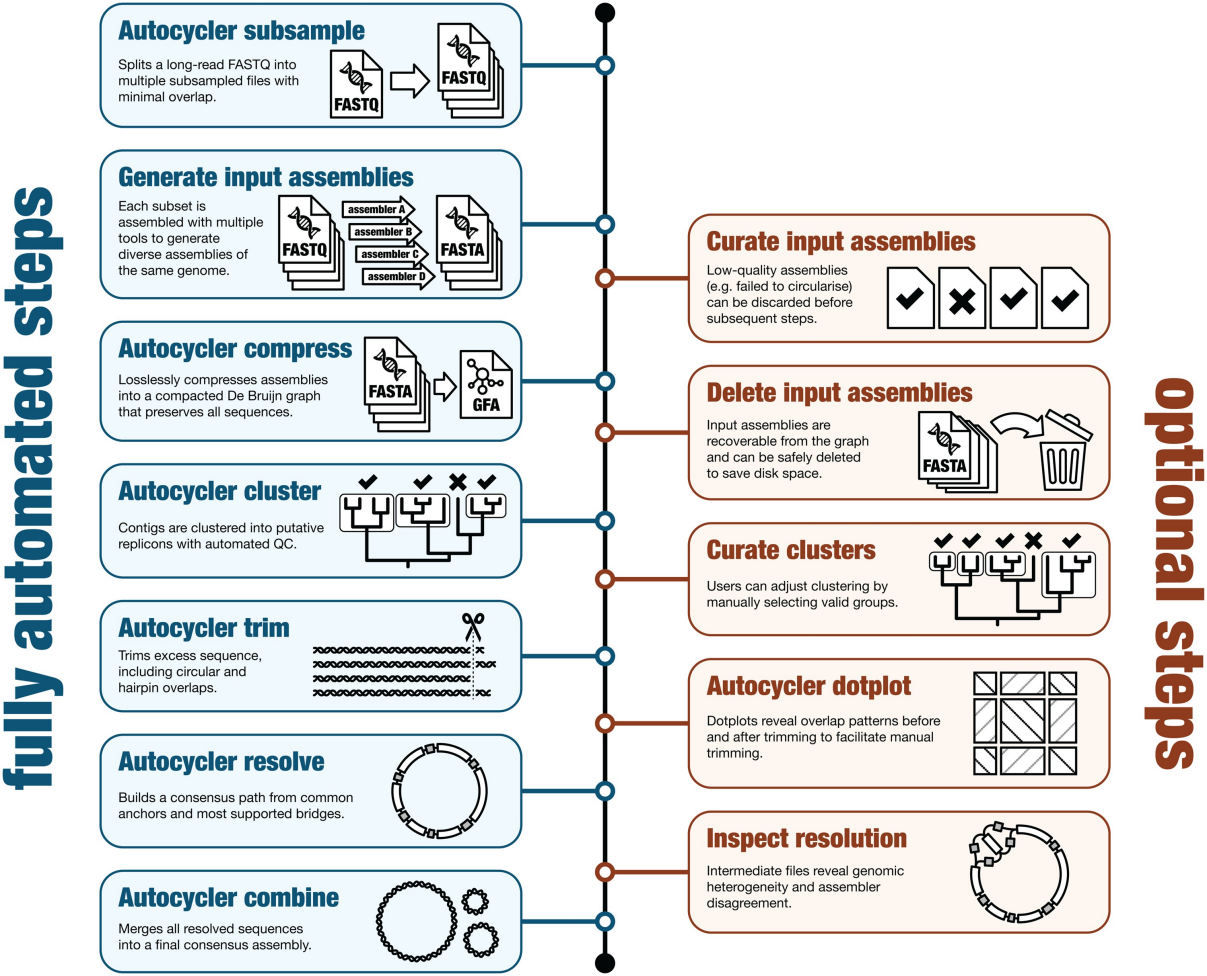
Overview of the Autocycler workflow. By following only the steps on the left, Autocycler can produce consensus genome assemblies with no human intervention. The optional steps on the right can be used when accuracy is critical or when Autocycler’s metrics (gathered using the autocycler table command) indicate potential issues.

Autocycler constructs a consensus bacterial genome assembly by combining multiple alternative assemblies of the same genome (Fig. 1). It is designed to run automatically and produces intermediate files and metrics for every step, allowing the process to be inspected or curated if needed. In addition to the main consensus assembly workflow, Autocycler includes commands to assist with upstream and downstream tasks.

The autocycler subsample command creates read subsets for generating input assemblies. By dividing a single long-read set into minimally overlapping subsets, users can generate assemblies that are more independent of one another. Additionally, the subsampling process reduces very high-depth read sets to medium-depth subsets, which often assemble more cleanly. Users can choose any number of subsets, but we recommend four (the default), which avoids excessive overlap between subsets while still enabling a diverse set of input assemblies.

Input assemblies should be generated using a range of long-read assemblers, as this diversity improves the robustness of the final consensus. For example, assembling four read subsets with eight different assemblers would yield 32 alternative assemblies of the same genome as input for Autocycler. The autocycler helper command provides a simple wrapper for several common tools. Ideally, input assemblies will have each replicon in the genome (e.g. chromosome or plasmid) assembled as a single contig. While some fragmentation is tolerated, Autocycler relies on the assumption that most input assemblies are complete. If all input assemblies are fragmented, Autocycler will not be able to produce a complete consensus. For challenging genomes, users may optionally curate the input assemblies (e.g. discard or repair incomplete contigs) before proceeding.

The autocycler compress command builds a compacted De Bruijn graph from the input assemblies. This graph substantially reduces disk usage, as shared regions across assemblies are collapsed together. Each input contig is recorded as a path through the graph, preserving its full sequence and allowing reconstruction with autocycler decompress. The graph representation provides an efficient structure for comparing and manipulating contigs in subsequent steps, such as clustering and trimming, since their graph paths can be compared directly without the need for sequence alignment.

In the autocycler cluster step, pairwise distances between contigs are calculated based on the overlap of their graph paths. These distances are used to build a UPGMA tree, which is divided into initial clusters by applying a fixed distance cutoff. Autocycler then refines these clusters by testing whether splitting them improves clustering balance (each input assembly contributing a single contig per cluster) and tightness (low intra-cluster distances). Quality control filters remove low-confidence clusters, such as those found in too few assemblies (likely spurious contigs) or those contained within other clusters (likely fragmented contigs or repetitive elements). The final result ideally includes one high-confidence cluster per replicon in the genome. Users may override the default clustering by inspecting the UPGMA tree and manually choosing which clusters to use. This can be helpful in challenging cases where input assemblies are inconsistent, e.g. to recover a small plasmid present in only a small number of inputs.

For each cluster, autocycler trim processes the input contigs to remove unwanted sequence. It looks for both circular overlaps, where the start of a contig overlaps with its end, and hairpin overlaps, where the start or end of a contig extends past the hairpin to the opposite strand. It also handles cases where small plasmids are fully duplicated within a single contig. After trimming, sequences with lengths that deviate too far from the cluster median are discarded, leaving a set of consistent contigs for consensus generation. Users may optionally run autocycler dotplot before and after trimming to visualize structural features and assess trimming outcomes.

The autocycler resolve command generates a consensus sequence for each cluster. It begins by identifying anchors: sequences that appear exactly once in each contig. These anchors serve as a scaffold for constructing bridges, which represent the most common paths between anchors in the input contigs. Autocycler first applies unambiguous bridges and then iteratively resolves ambiguous cases by selecting the most supported paths, ideally producing a single consensus sequence for the cluster. In cases of structural heterogeneity, such as phase-variable loci or assembly inconsistencies, Autocycler includes intermediate output for optional user inspection. When a cluster fails to fully resolve, the user can inspect its graph in Bandage (Wick *et al.* 2015) and use the autocycler clean command to manually remove or duplicate segments, often useful for the ends of linear plasmids.

Once each cluster has been resolved, the autocycler combine command merges them into a final consensus assembly in both FASTA and GFA formats. Autocycler also produces detailed metrics at every step of the pipeline, saved in YAML format (both human- and machine-readable). The autocycler table command can be used to gather metrics from many assemblies, making it easy to track success and identify samples that require further attention.

Compared to its predecessor Trycycler, Autocycler is designed to run to completion without user intervention. Two steps in the Trycycler pipeline typically require manual input: defining clusters and reconciling sequences into a consensus. Autocycler improves the former by automatically identifying clusters using UPGMA and quality control heuristics. For the latter, it replaces Trycycler’s sequence-alignment-based reconciliation (which often failed in the presence of low-quality contigs) with a more robust De Bruijn graph-based approach. Even for steps that did not require user input in Trycycler, Autocycler is faster due to its efficient data structures and algorithms. Since Autocycler also supports manual curation when needed, it is now the recommended tool for long-read consensus bacterial genome assembly.

Autocycler is implemented in Rust, deterministic, and resource-efficient. Most of the computational time in an Autocycler workflow is spent generating input assemblies. Autocycler itself typically completes in minutes and requires only modest resources. It runs on both Linux and macOS, although Linux is preferred due to broader compatibility with long-read assemblers. Extensive documentation, including illustrated examples and guidance for manual curation, is available online at github.com/rrwick/Autocycler/wiki.

## 3 Evaluation

### 3.1 Methods

Long-read sequencing of 84 diverse bacterial isolates was performed using an Oxford Nanopore Technologies PromethION 2 Solo with the Rapid Barcoding Kit 96 V14 (SQK-RBK114.96). Reads were basecalled with Dorado v0.9.5 (github.com/nanoporetech/dorado) using the sup@v5.0.0 model and filtered to retain reads with mean quality ≥10. Five isolates were selected, each from a different genus: *Enterobacter hormaechei*, *Klebsiella pneumoniae*, *Listeria innocua*, *Providencia rettgeri*, and *Shigella flexneri*. Short-read Illumina sequencing was available for all samples and was used to polish the reference genomes (Table S1, available as supplementary data at *Bioinformatics* online). Selection was based on high read depth (>300×) and preliminary assessments showing no evidence of heterogeneity or divergence between the Illumina and ONT datasets.

For each genome, we followed our previously published method to generate a high-accuracy reference assembly (Wick *et al.* 2023). Briefly, the ONT reads were assembled with Trycycler v0.5.5 (Wick *et al.* 2021) and the resulting genome was polished using Medaka v2.0.1 (github.com/nanoporetech/medaka), Polypolish v0.6.0 (Wick and Holt 2022), and Pypolca v0.3.1 (Bouras *et al.* 2024b). The resulting assemblies were highly accurate and used as ground truth.

ONT reads for each genome were divided into six non-overlapping 50× subsets for a total of 30 read sets. Each read set was assembled using the following long-read assemblers: Canu v2.3 (Koren *et al.* 2017), Flye v2.9.5 (Kolmogorov *et al.* 2019), hifiasm v0.25.0 (Cheng *et al.* 2021), LJA v0.2 (Bankevich *et al.* 2022), metaMDBG (a.k.a. nanoMDBG) v1.1 (Benoit *et al.* 2024), miniasm v0.3 (Li 2016), Myloasm v0.1.0 (github.com/bluenote-1577/myloasm), NECAT v0.0.1 (Chen *et al.* 2021a), NextDenovo v2.5.2 (Hu *et al.* 2024), Raven v1.8.3 (Vaser and Šikić 2021), and wtdbg2 v2.5 (Ruan and Li 2020). Each of these tools was run via the autocycler helper command, which included low-depth contig removal and extra processing for Canu (overlap-trimming and repeat/bubble removal), miniasm [polishing with Minipolish (github.com/rrwick/Minipolish)], and NextDenovo [polishing with NextPolish (Hu *et al.* 2020)].

In addition, we assembled each read set with the long-read assembly pipelines Dragonflye v1.2.1 (github.com/rpetit3/dragonflye) and Hybracter v0.11.2 (Bouras *et al.* 2024a) and the consensus assembly tool MAECI (Lang 2022) (commit f1eb3d7). For Autocycler v0.5.1, we produced an automated assembly (using its autocycler_full.sh script) and a manually curated assembly. All assembly commands were run through GNU Time to quantify runtime and memory, using 32 threads on a system with dual AMD EPYC 7742 CPUs and 503 GB RAM.

We also attempted to evaluate MAC2.0 (Tang *et al.* 2019), but it did not perform correctly on complete bacterial genomes, producing outputs with duplicated sequences. Other consensus assembly tools, such as quickmerge (Chakraborty *et al.* 2016) and Metassembler (Wences and Schatz 2015), are older and were primarily designed to improve contiguity in fragmented eukaryotic assemblies. These tools are not suitable for refining complete bacterial genomes from long-read data and were therefore excluded from this comparison (Alhakami *et al.* 2017).

Each assembly was compared to its corresponding ground-truth reference using a custom script (assess_assembly.py) that aligns the assembly to the reference sequence with minimap2 v2.28 (Li 2018) and quantifies accuracy metrics including sequence errors (substitutions and indels), missing bases and extra bases. We also assessed assembly accuracy with Inspector v1.3.1 (Chen *et al.* 2021b) and CRAQ v1.0.9 (Li *et al.* 2023), which evaluate assemblies based on read alignments rather than a reference, and BUSCO v6.0.0 (Tegenfeldt *et al.* 2025), which evaluates assemblies based on the presence of expected single-copy genes.

To evaluate Autocycler’s performance on challenging datasets, we performed two additional tests. In the low-depth test, the *L. innocua* genome was assembled across a range of read depths (1× to 50×) and assessed using the assess_assembly.py script. In the mixed-genome test, we combined reads from the *E. hormaechei* and *K. pneumoniae* genomes at varying ratios (total depth fixed at 100×) and evaluated the resulting assemblies based on contig completeness and genome of origin. Full commands for all analyses are provided in the supplementary data.

### 3.2 Results

**Figure 2.**
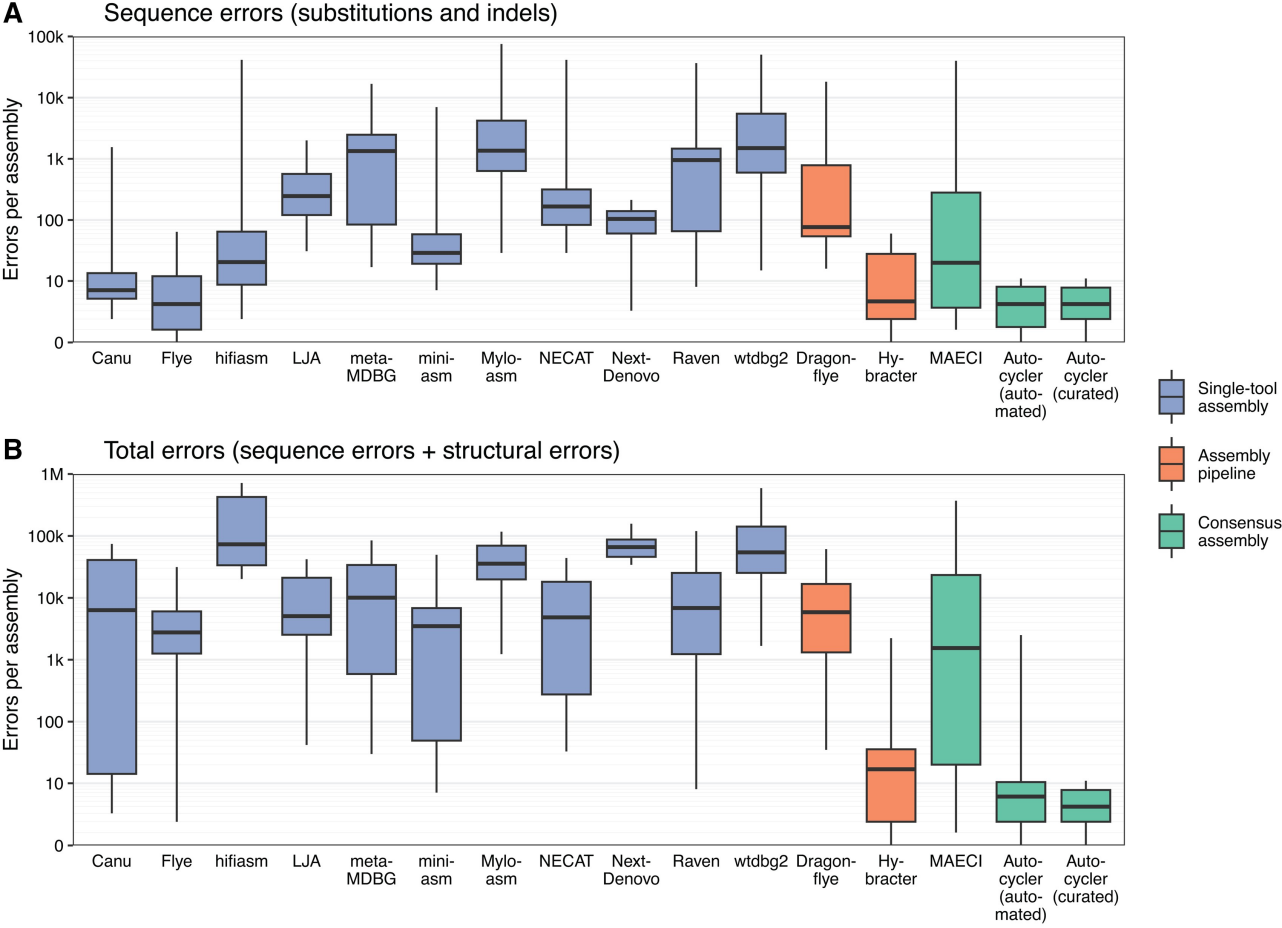
Assembler benchmarking results from the assess_assembly.py script. (A) Sequence errors (substitutions and indels); (B) Sequence errors and structural assembly errors (missing and extra bases). Lower values indicate better accuracy. Results are coloured by category: individual long-read assembly tools (blue), long-read assembly pipelines (orange), and consensus assembly tools (green). Autocycler results are shown separately for automated and manually curated assemblies. Boxplot whiskers extend to the minimum and maximum values. The *y*-axes use a pseudo-logarithmic scale that accommodates zeros. See Table S2 and Fig. S1 available as supplementary data at *Bioinformatics* online, for results broken down by error type and Table S3, available as supplementary data at *Bioinformatics* online, for mean values per assembler.

Among the single-tool assemblers, Canu and Flye consistently produced the fewest sequence-level errors, typically with fewer than 10 substitutions and indel errors per assembly (Fig. 2A and Fig. S1 and Table S2, available as supplementary data at *Bioinformatics* online). All other long-read assemblers had higher error rates. Across all single-tool assemblers, structural inaccuracies were common (Fig. 2B and Fig. S1, available as supplementary data at *Bioinformatics* online), with assemblies often missing genomic elements (e.g. small plasmids) or containing spurious extra sequence (e.g. duplicated ends of circular contigs).

Of the long-read assembly pipelines, Hybracter outperformed Dragonflye. By integrating Plassembler (Bouras *et al.* 2023), Hybracter improves plasmid recovery and avoids structural errors such as duplication of plasmids. However, for the *E. hormaechei* and *K. pneumoniae* genomes, Hybracter showed elevated error rates in large plasmids, likely due to errors introduced by Unicycler (Wick *et al.* 2017) within Plassembler (Table S4, available as supplementary data at *Bioinformatics* online). Dragonflye performed worse than Flye alone, likely due to its default use of Racon polishing (instead of Flye’s internal polisher) and the --nano-raw option which is suboptimal for modern ONT reads. With adjusted parameters (--racon 0--opts ’-i 1’--nanohq), Dragonflye’s performance matched that of Flye (Table S5, available as supplementary data at *Bioinformatics* online).

MAECI was the only consensus assembly tool tested apart from Autocycler. Despite incorporating three input assemblers (Canu, Flye, and wtdbg2), MAECI did not consistently outperform Canu or Flye.

When run in an automated manner, Autocycler produced assemblies with the lowest sequence error counts of any method (median: 4 errors per assembly, range: 0–11). It was also structurally accurate in most cases, successfully recovering all replicons for four of the five genomes. This is in part because autocycler_full.sh uses Plassembler when generating input assemblies. However, the smallest plasmid of the *E. hormaechei* genome (2.5 kbp) was occasionally missed. In manually curated runs, the missing plasmid could be identified by inspecting the clustering tree (output by autocycler cluster) and included in the final assembly by overriding the default clustering. These curated Autocycler assemblies had no structural errors reported by assess_assembly.py, Inspector, CRAQ, or BUSCO (Table S2 and Figs S1–S4, available as supplementary data at *Bioinformatics* online).

Autocycler was the slowest assembly method tested, with a median runtime of 1 h 44 min (Table S2 and Fig. S5, available as supplementary data at *Bioinformatics* online), due to the time required to generate multiple input assemblies. Raven was the fastest, with a median runtime of 30 s per assembly. Autocycler had the second-highest peak RAM usage after NECAT, with a median of 11.3 GB, determined by the most memory-intensive assembler in its pipeline (NECAT). metaMDBG was the most memory-efficient, with a median of 1.2 GB.

In the low-depth test, Autocycler produced high-quality assemblies (no structural errors and six or fewer sequence errors) down to a depth of 23× (Table S6 and Fig. S6, available as supplementary data at *Bioinformatics* online). Between 13× and 22×, results were more variable, with some assemblies failing and others exhibiting major structural errors. At depths of 12× and below, all assemblies failed. In the mixed-genome test, Autocycler assemblies remained uncontaminated when the secondary genome was present at less than ∼0.5× depth (Table S7 and Fig. S7, available as supplementary data at *Bioinformatics* online). At contamination levels between ∼0.5× and ∼20×, Autocycler assemblies increasingly included plasmids from the secondary genome—first high-copy-number small plasmids, then low-copy-number large plasmids. At contamination levels >20×, the results became erratic, with assemblies sometimes including both chromosomes or neither. Across all tests, Autocycler produced only complete circular contigs, with a single exception at a 69:31 mixture where the *K. pneumoniae* chromosome was fragmented.

## 4 Discussion and conclusions

Consensus assembly offers a clear accuracy advantage over single-tool assembly. In our benchmarking, even the best-performing individual assemblers (Canu and Flye) consistently made avoidable errors. Consensus approaches mitigate such issues by averaging over multiple inputs, reducing both small-scale errors and structural inaccuracies. Trycycler (Wick *et al.* 2021) provided a robust framework for generating consensus bacterial genome assemblies, but it requires substantial user intervention, limiting its scalability. Autocycler brings the benefits of consensus assembly into an automated workflow, enabling accurate bacterial genome assembly at scale.

Autocycler does not guarantee perfect results, as its consensus reflects the input assemblies. If the inputs are fragmented (e.g. due to a repeat longer than the read length), Autocycler cannot produce a fully resolved consensus. When most inputs share the same error, that error can persist into the final assembly. In our evaluation, there were typically fewer than 10 sequence errors (substitutions and indels) per Autocycler assembly (Table S8, available as supplementary data at *Bioinformatics* online), most commonly homopolymer-length errors resulting from systematic basecalling issues in ONT reads (Sereika *et al.* 2022). These errors are often inconsistent between runs, as they tend to occur at ambiguous sites where input assemblies disagree (e.g. with roughly equal support for two alternatives), which is why the curated Autocycler assemblies in this study did not always have the same error count as the automated Autocycler assemblies. The frequency of these errors depends on factors such as pore type (R10.4.1 is more accurate than R9.4.1), basecalling model (sup is more accurate than hac or fast), and bacterial strain. To address these errors, short-read polishing can be applied after Autocycler, yielding hybrid assemblies with maximal accuracy (Bouras *et al.* 2024b).

The only structural error observed in automated Autocycler assemblies in this study was the omission of a small plasmid from the *E. hormaechei* genome. Small plasmids are a common point of failure for long-read assemblers (Johnson *et al.* 2023, Lerminiaux *et al.* 2024). We included Plassembler among the input assemblers to improve small-plasmid recovery, but it often failed to circularize this plasmid due to a homopolymer sequence, leading to its exclusion during Autocycler’s clustering step. However, Autocycler supports manual intervention at key steps in its pipeline, and in this case, manual review of the clustering enabled recovery of this plasmid. This flexible design allows Autocycler to function as both a scalable automated tool and a framework for high-accuracy curated reference genome assembly.

The set of input assemblers used with Autocycler is flexible and can be tailored to the user’s needs. For example, Canu is a good choice when accuracy is a priority but may be excluded when faster runtimes are needed. Using multiple assemblers increases robustness, as no single tool performs best across all datasets. In this study, we used the autocycler_full.sh script provided with Autocycler, which runs eight assemblers: Canu, Flye, metaMDBG, miniasm, NECAT, NextDenovo, Plassembler, and Raven. It excludes Myloasm and wtdbg2, which tend to produce high sequence error rates; hifiasm, which frequently generates extra contigs; LJA, which is recommended only for PacBio HiFi reads; and Hybracter, which internally runs both Flye and Plassembler, already included separately. This script is in Autocycler’s pipelines directory, which invites users to contribute alternative pipelines, e.g. using different assemblers, parameters, or workflow managers such as Nextflow (Di Tommaso *et al.* 2017). We have not conducted a systematic evaluation of which assembler combinations perform best for different genome types or sequencing conditions, and this remains an important direction for future work.

Although not evaluated in this study, linear replicons pose additional challenges for genome assembly. Assemblers may erroneously extend hairpin ends or terminate open ends inconsistently. While Autocycler includes logic to detect and trim hairpin overlaps, full resolution of linear sequences still frequently requires manual intervention via the autocycler clean command. Improved support for such cases remains an area for improvement for both long-read assemblers and Autocycler.

Structural heterogeneity in the input assemblies is collapsed by Autocycler, which resolves each cluster by selecting the most supported path. As a result, assemblies from heterogeneous genomes will reflect the most common structural configuration present in the input assemblies. Significant heterogeneity may be visible in a cluster’s intermediate output file (4_merged.gfa), but a more comprehensive characterization is better performed after assembly using a structural variant caller such as Sniffles (Smolka *et al.* 2024).

While Autocycler is designed for haploid prokaryotic isolate genomes, it may also be applicable in other contexts, with caveats. For eukaryotic genomes, Autocycler’s assumption that input assemblies are complete makes it unsuitable for chromosomes that cannot be assembled end to end. Phased diploid assemblies also pose challenges, as both haplotypes are likely to be grouped together during clustering, so users would need to separate haplotypes and run Autocycler on each independently. Repetitive ends of linear chromosomes are difficult for Autocycler to resolve and would likely require manual finishing. For metagenomes, assemblies are generally too fragmented for direct use with Autocycler, but if a metagenome contains high-depth components that assemble completely, these can be isolated and processed with Autocycler as individual genomes.

Autocycler is open-source, well documented and easy to install. It requires only modest system resources (excluding input assembly generation) and provides intermediate outputs to support transparency and manual curation. It fills a key gap in the current assembly tool landscape: existing consensus tools either underperform or do not scale, while long-read assembly pipelines rely on a single assembler and inherit its limitations. Because it relies on multiple inputs, an Autocycler-based pipeline is more computationally intensive than using a single assembler, but it usually yields better assemblies. We therefore recommend Autocycler for long-read bacterial genome projects where maximum assembly accuracy is required.

## Supplementary Material

btaf474_Supplementary_Data

## Data Availability

The data underlying this article are available at github.com/rrwick/Autocycler-paper and archived on Zenodo (doi: 10.5281/zenodo.16916187). Assemblies, reference genomes and read sets used in the analysis are available at figshare.unimelb.edu.au/projects/Autocycler/247142. Conflict of interest: The authors declare that there are no conflicts of interest.
