# Supplementary material for: Autocycler: long-read consensus assembly for bacterial genomes
Source: Bioinformatics. 2025 Aug 28;41(9):btaf474. doi: 10.1093/bioinformatics/btaf474 (PMC12460055; doi:10.1093/bioinformatics/btaf474)
Supplement: btaf474_Supplementary_Data [file btaf474_supplementary_data.zip › autocycler_supp_figures.pdf]

This document contains the supplementary figures for the manuscript *Autocycler: long-read consensus assembly for bacterial genomes*.

Figures S1–S5 present boxplots summarising results by tool. Results are coloured by category: individual long-read assembly tools (blue), long-read assembly pipelines (orange) and consensus assembly tools (green). Each boxplot shows the median (central line), interquartile range (box), and full data range (whiskers, no values are treated as outliers). To accommodate the wide range of values, many plots use a pseudo-logarithmic transformation on the y-axis, which behaves like a log scale but includes zero.

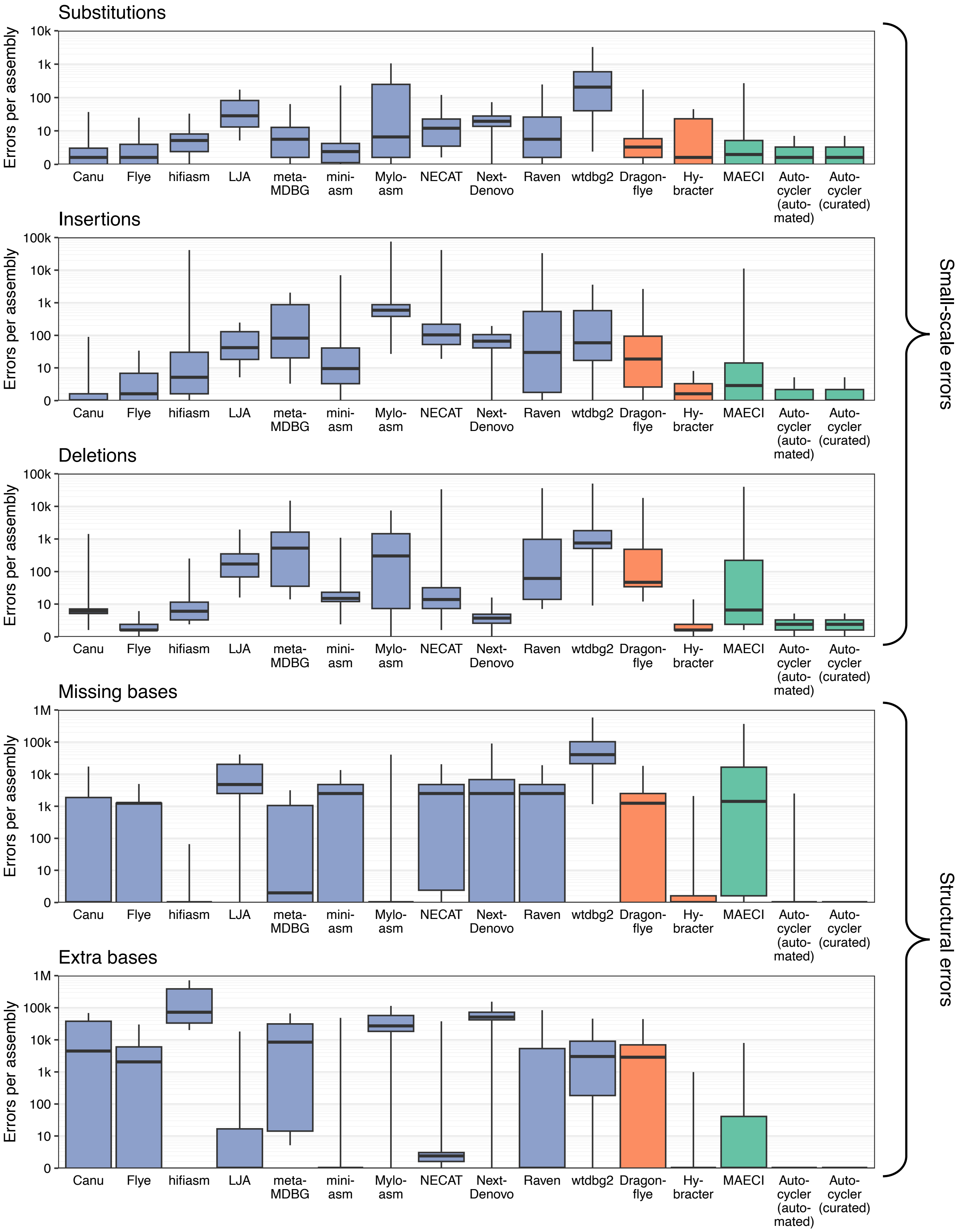

Figure S1: Detailed benchmarking results from the `assess_assembly.py` script, showing individual error types. Substitutions: mismatched bases between the assembly and reference. Insertions: extra bases present in the assembly but not in the reference (within aligned regions). Deletions: bases missing from the assembly but present in the reference (within aligned regions). Missing bases: unaligned regions of the reference. Extra bases: unaligned regions in the assembly or duplicated content. Lower values indicate better accuracy.

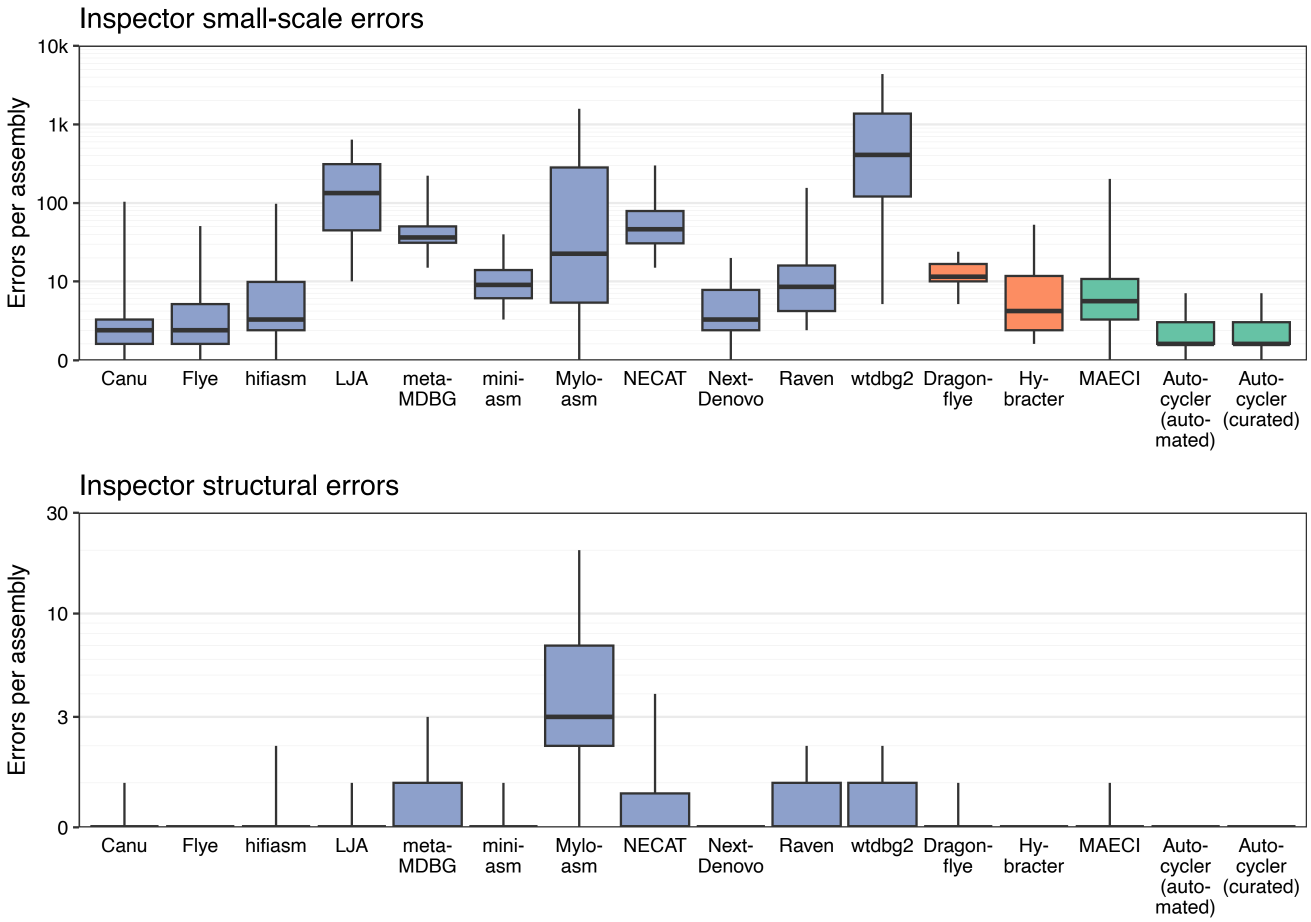

Figure S2: Assembly benchmarking results from Inspector, which evaluates assemblies using long-read alignments. Inspector small-scale errors: <50 bp including base substitutions, small expansions and small collapses. Inspector structural errors: ≥50 bp including large expansions, large collapses and inversions. Lower values indicate better accuracy.

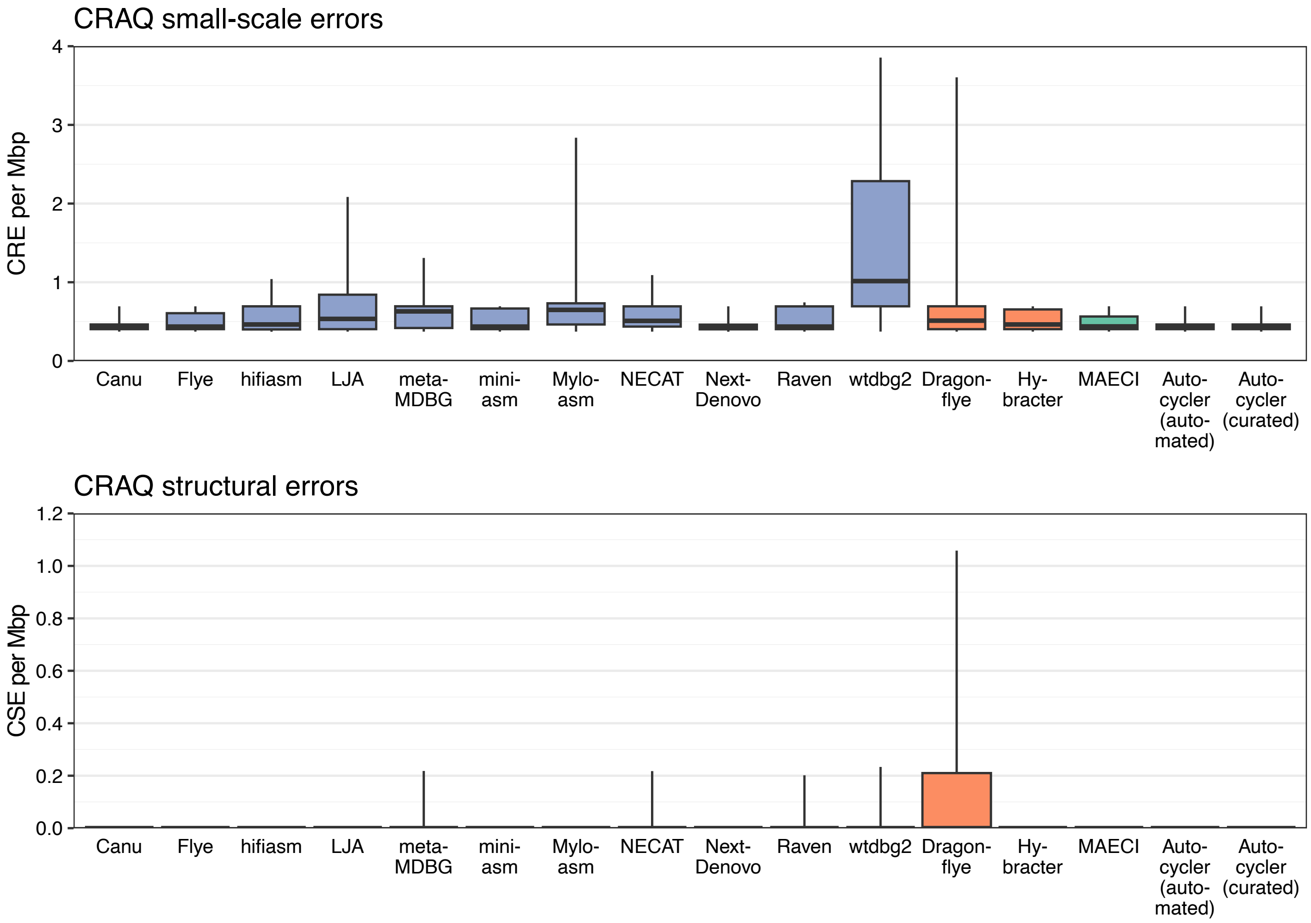

Figure S3: Assembly benchmarking results from CRAQ, which evaluates assemblies using both short- and long-read alignments. CRAQ small-scale errors: clip-based regional errors (CRE), defined as regions where short reads are clipped or absent but long reads align fully. CRAQ structural errors: clip-based structural errors (CSE), defined as regions where both short and long reads exhibit clipping near the same breakpoint. Lower values indicate better assembly accuracy.

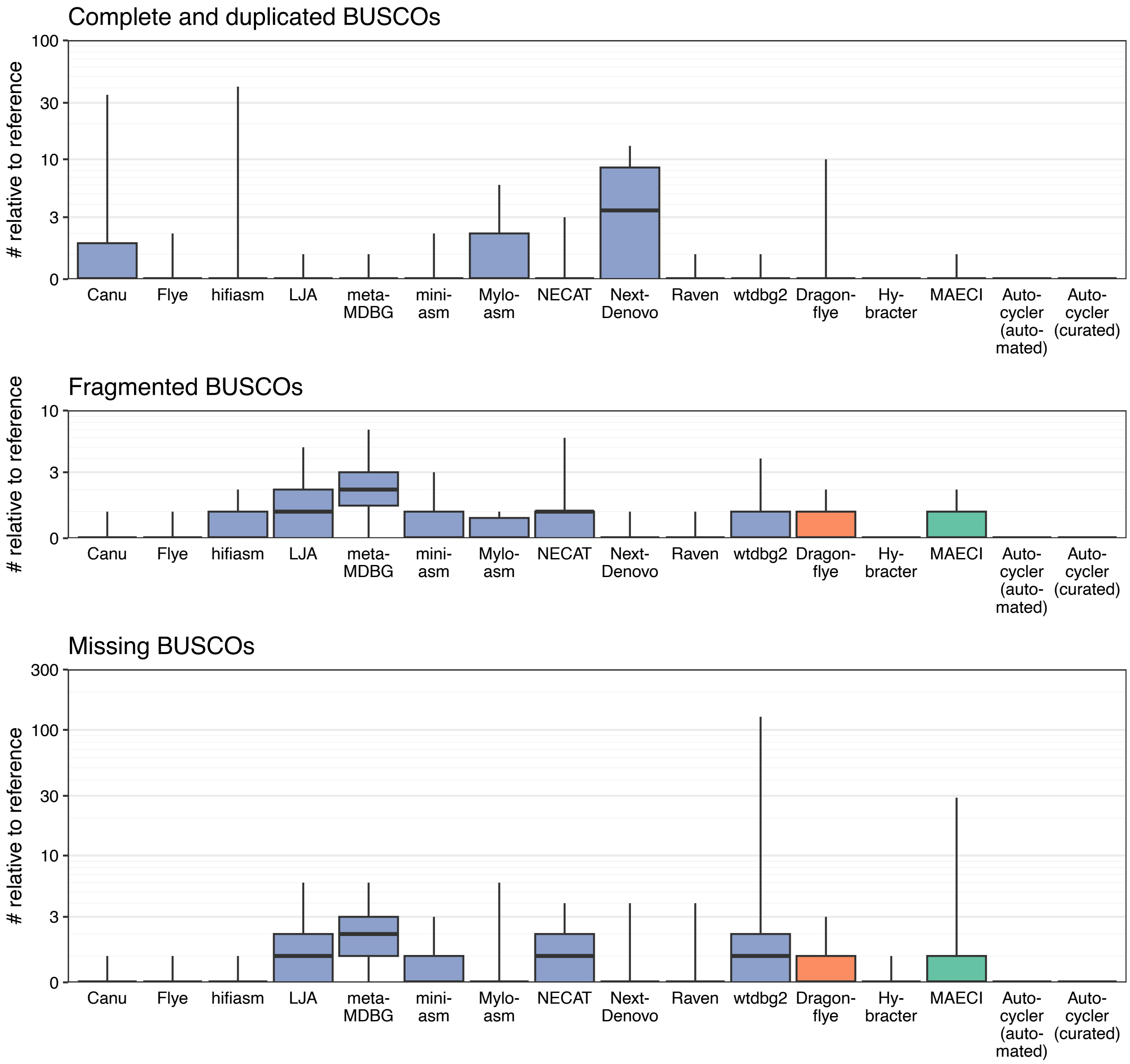

Figure S4: Assembly benchmarking results from BUSCO, which evaluates assemblies using expected single-copy orthologous genes. Complete and duplicated BUSCOs: genes found more than once in the assembly, indicating possible assembly redundancy. Fragmented BUSCOs: partially recovered genes, suggesting incomplete or erroneous assembly in those regions. Missing BUSCOs: genes expected for the lineage but not found in the assembly. All values represent the absolute difference between each BUSCO category in the assembly and the corresponding reference genome (i.e. |assembly − reference|). Lower values indicate better accuracy.

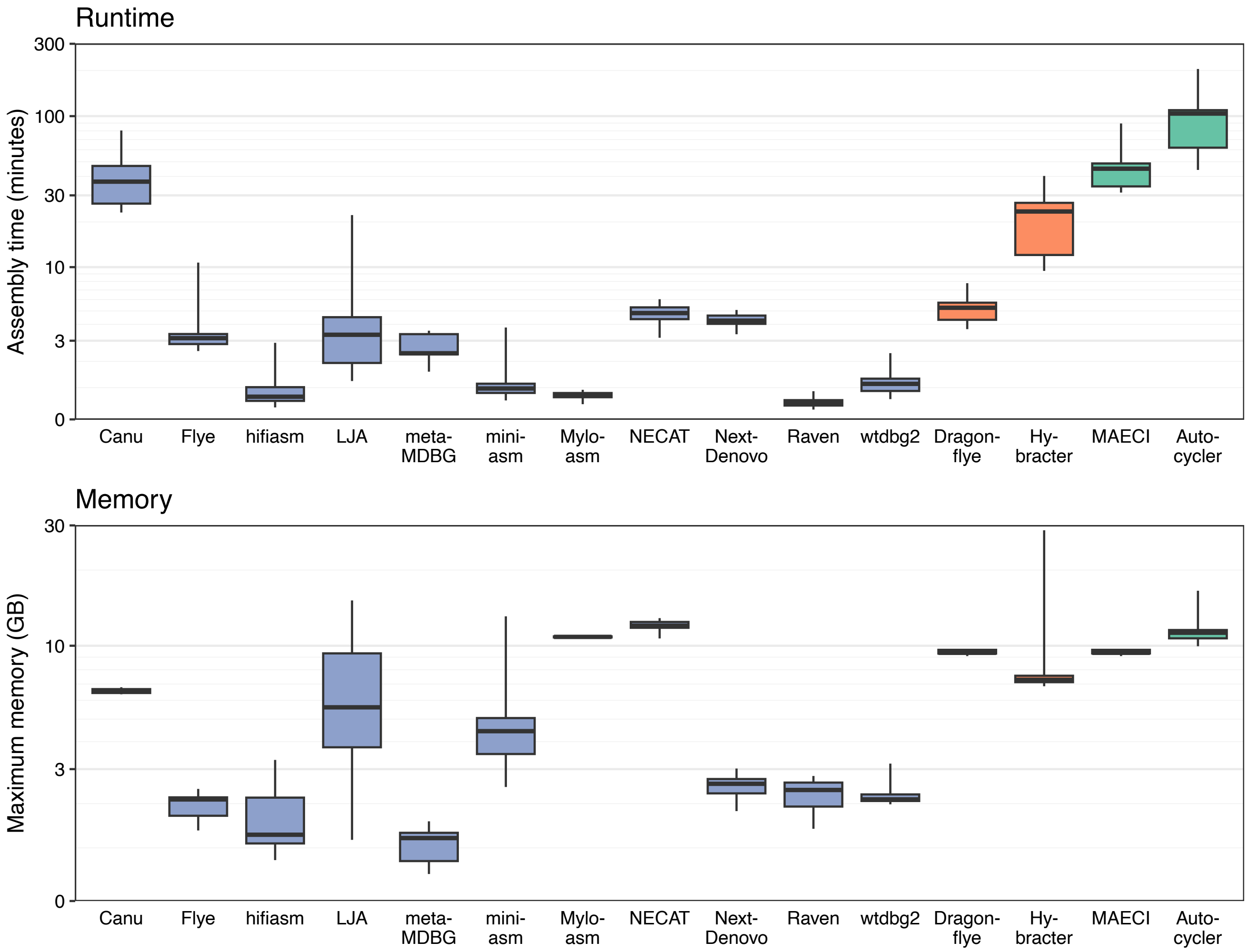

Figure S5: Runtime and peak memory usage as reported by GNU Time. Lower values indicate better computational performance. Autocycler results include the generation of input assemblies.

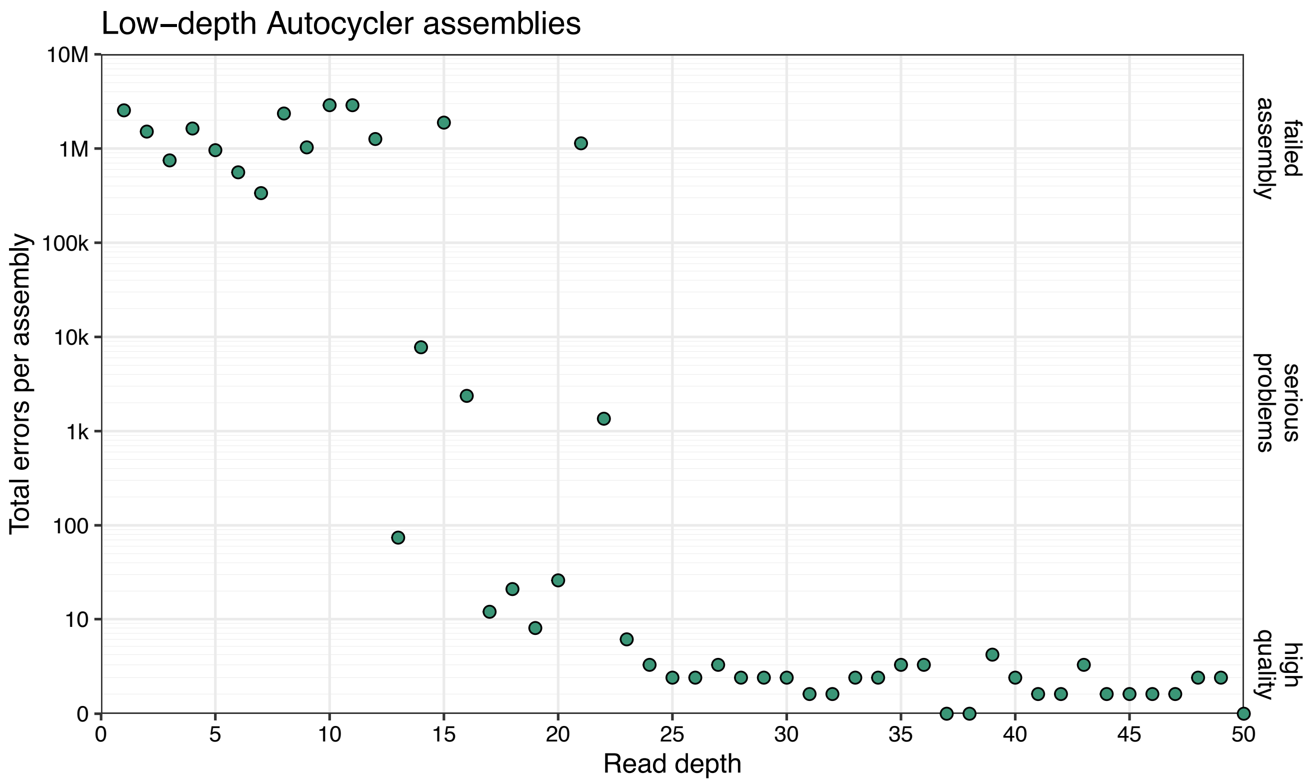

Figure S6: Autocycler assembly accuracy at low read depths using the *Listeria innocua* dataset. Each point represents the total number of sequence and structural errors, as reported by `assess_assembly.py`. Lower values indicate better accuracy. Autocycler produced accurate assemblies at 23× depth and higher but typically failed below this threshold. With default parameters, Autocycler requires at least 25× read depth, so adjustments were made to the subsampling, compression and clustering steps to enable assemblies at very low depths; see supplementary methods for full details.

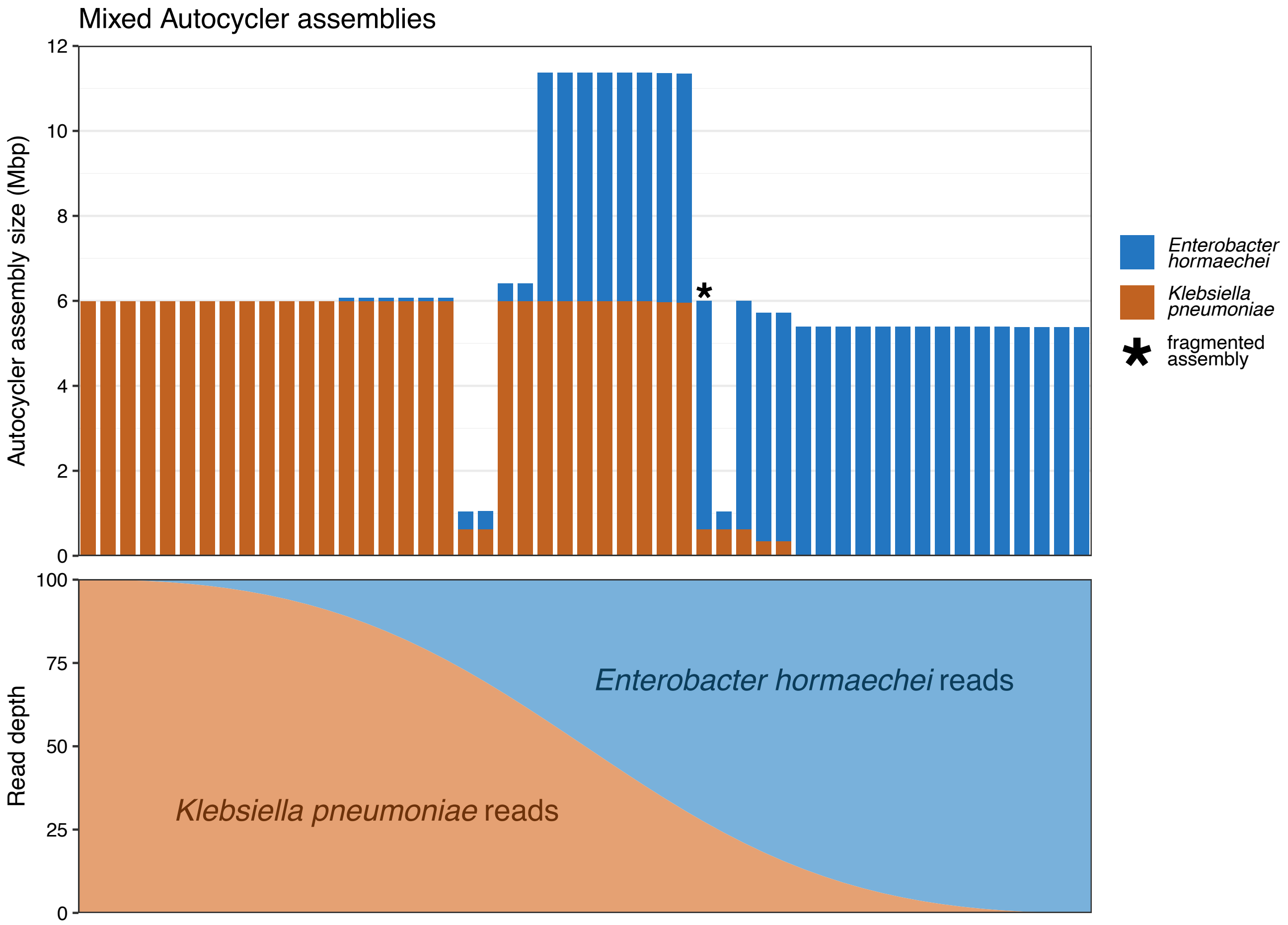

Figure S7: Assembly accuracy of Autocycler on mixed-genome datasets containing varying proportions of *Enterobacter hormaechei* and *Klebsiella pneumoniae* reads. The total read depth was fixed at 100×, with relative proportions of the two genomes ranging from 100% *K. pneumoniae* to 100% *E. hormaechei*. Each bar shows the number of bases in the final Autocycler assembly categorised by their source genome. For very low levels of contamination, Autocycler assemblies remained uncontaminated. As contaminant depth increased, high-copy-number plasmids from the minor genome began to appear (at ∼0.5× depth), followed by larger plasmids (at ∼12× depth). At very high levels of contamination (>20×), results became more variable, with some assemblies containing only plasmids or containing chromosomes from both genomes. All Autocycler assemblies consisted entirely of complete circular contigs, except for a single case where the *K. pneumoniae* chromosome was fragmented.
